# Supplementary material for: Analysis of the Behavioral Change and Utility Features of Electronic Activity Monitors
Source: Technologies (Basel). Author manuscript; Available in PMC 2025 Jan 28. (PMC11774501; doi:10.3390/technologies8040075)
Supplement: Additional File 1 [file NIHMS2016016-supplement-Additional_File_1.doc]

**Table 1. Wearables reviewed**

|  | **ZHL** | **MC** | **GR** |
| --- | --- | --- | --- |
| **Apple Watch** |  |  |  |
| **Fitbit Versa 2** |  | **3/19** | **2/19** |
| **Fitbit Charge 3** |  | **2/06** | **3/04** |
| **Fitbit Ionic** |  | **2/20** | **3/18** |
| **Garmin Vivomove HR** |  | **2/20** | **3/18** |
| **Garmin Vivosmart 4** |  | **2/06** | **3/04** |
| **Amazefit Bip** |  | **3/26** | **2/19** |
| **Galaxy Watch Active** |  | **3/05** | **3/25** |
| **Withings Steel HR** |  | **3/05** | **3/25** |

**First, Second (blinded), Third (unblinded)**

**Table 2**. Behavior Change Techniques from the hierarchical taxonomy

| **BCT** | **Apple Watch** | **Fitbit Versa 2** | **Fitbit Charge 3** | **Fitbit Ionic (Addidas)** | **Garmin Vivomove HR** | **Garmin Vivosmart 4** | **Amazefit Bip** | **Galaxy Watch Active** | **Withings Steel HR** | **TOTAL** |
| --- | --- | --- | --- | --- | --- | --- | --- | --- | --- | --- |
| 1. Goals and planning |  |  |  |  |  |  |  |  |  |  |
| 1.1 Goal setting (behavior) | X | X | X | X | X | X | X | X | X | **9** |
| 1.2 Problem solving |  | - | - | - |  |  |  |  |  | **0** |
| 1.3 Goal setting (outcome) |  | X | X | X | X | X | X | X |  | **6** |
| 1.4 Action planning |  |  |  |  |  |  |  |  |  | **0** |
| 1.5 Review behavior goal(s) | X | X | X | X | X | X | X | X | X | **9** |
| 1.6 Discrepancy between current behavior and goal | X | X | X | X | X | X | X | X | X | **9** |
| 1.7 Review outcome goal(s) |  | X | X | X |  |  | X | X |  | **5** |
| 1.8 Behavioral contract | X | X | X | X |  |  | X | X | X | **7** |
| 1.9 Commitment | X | X | X | X | X | X |  | X | X | **8** |
| 2. Feedback and monitoring |  |  |  |  |  |  |  |  |  |  |
| 2.1 Monitoring of behavior by others without feedback |  |  |  |  |  |  |  |  |  | **0** |
| 2.2 Feedback on behavior | X | X | X | X | X | X | X | X | X | **9** |
| 2.3 Self-monitoring of behavior | X | X | X | X | X | X | X | X | X | **9** |
| 2.4 Self-monitoring of outcome(s) of behavior |  | X | X | X | X | X | X |  |  | **6** |
| 2.5 Monitoring of outcome(s) of behavior without feedback |  |  |  |  | X | X |  |  |  | **2** |
| 2.6 Biofeedback | X | X | X | X | X | X | X | X | X | **9** |
| 2.7 Feedback on outcome(s) of behavior |  | X | X | X |  |  | X |  |  | **4** |
| 3. Social support |  |  |  |  |  |  |  |  |  |  |
| 3.1. Social support (unspecified) | X | X | X | X | X | X | X | X | X | **9** |
| 3.2. Social support (practical) |  |  |  |  |  |  |  |  |  | **0** |
| 3.3. Social support (emotional) |  | X | X | X | X | X | X |  |  | **6** |
| 4. Shaping knowledge |  |  |  |  |  |  |  |  |  |  |
| 4.1 Instruction on how to perform the behavior |  | X | X | X | X | X |  | X | X | **7** |
| 4.2 Information about antecedents |  | X | X | X | X | X |  |  |  | **5** |
| 4.3 Re-attribution |  |  |  |  |  |  |  |  |  | **0** |
| 4.4 Behavioral experiments |  |  |  |  | X | X |  |  |  | **2** |
| 5. Natural consequences |  |  |  |  |  |  |  |  |  |  |
| 5.1 Information about health consequences |  | X | X | X | X | X |  |  |  | **5** |
| 5.2 Salience of consequences |  |  |  |  |  |  |  |  |  | **0** |
| 5.3 Information about social and environmental consequences |  |  |  |  |  |  |  |  |  | **0** |
| 5.4 Monitoring of emotional consequences |  |  |  |  |  |  |  |  |  | **0** |
| 5.5 Anticipated regret |  | X | X | X |  |  |  |  |  | **3** |
| 5.6 Information about emotional consequences |  | X | X | X |  |  |  |  |  | **3** |
| 6. Comparison of behavior |  |  |  |  |  |  |  |  |  |  |
| 6.1 Demonstration of the behavior |  | X | X | X |  |  |  |  |  | **3** |
| 6.2 Social comparison | X | X | X | X | X | X | X | X | X | **9** |
| 6.3 Information about others’ approval |  | X | X | X |  |  |  |  |  | **3** |
| 7. Associations |  |  |  |  |  |  |  |  |  |  |
| 7.1 Prompts/cues | X | X | X | X | X | X | X | X | X | **9** |
| 7.2 Cue signaling reward |  | X | X | X | X | X | X |  |  | **6** |
| 7.3 Reduce prompts/cues |  |  |  |  | X | X |  |  |  | **2** |
| 7.4 Remove access to the reward |  |  |  |  |  |  |  |  |  | **0** |
| 7.5 Remove aversive stimulus |  |  |  |  |  |  |  |  |  | **0** |
| 7.6 Satiation |  |  |  |  |  |  |  |  |  | **0** |
| 7.7 Exposure |  |  |  |  |  |  |  |  |  | **0** |
| 7.8 Associative learning |  |  |  |  |  |  |  |  |  | **0** |
| 8 Repetition and substitution |  |  |  |  |  |  |  |  |  |  |
| 8.1 Behavioral practice/rehearsal |  | - | - | - |  |  |  | X |  | **1** |
| 8.2 Behavior substitution |  | X | X | X |  |  |  | X |  | **4** |
| 8.3 Habit formation |  | X | X | X | X | X | X | X | X | **8** |
| 8.4 Habit reversal |  |  |  |  | X | X |  |  |  | **2** |
| 8.5 Overcorrection |  |  |  |  |  |  |  |  |  | **0** |
| 8.6 Generalisation of target behavior |  |  |  |  |  |  |  |  |  | **0** |
| 8.7 Graded tasks | X | X | X | X | X | X |  |  |  | **6** |
| 9. Comparison of outcomes |  |  |  |  |  |  |  |  |  |  |
| 9.1 Credible source |  | X | X | X |  |  |  |  |  | **3** |
| 9.2 Pros and cons |  |  |  |  |  |  |  |  |  | **0** |
| 9.3 Comparative imagining of future outcomes |  |  |  |  | X | X |  |  |  | **2** |
| 10. Reward and threat |  |  |  |  |  |  |  |  |  |  |
| 10.1 Material incentive (behavior) |  |  |  |  |  |  |  |  |  | **0** |
| 10.2 Material reward (behavior) |  |  |  |  |  |  |  |  |  | **0** |
| 10.3 Non-specific reward | X | X | X | X | X | X | X | X | X | **9** |
| 10.4 Social reward |  | X | X | X |  |  | X |  |  | **4** |
| 10.5 Social incentive |  |  |  |  |  |  |  | X |  | **1** |
| 10.6 Non-specific incentive | X | X | X | X | X | X |  |  | X | **7** |
| 10.7 Self-incentive |  |  |  |  |  |  |  |  |  | **0** |
| 10.8 Incentive (outcome) | X | X | X | X |  |  |  |  |  | **4** |
| 10.9 Self-reward |  |  |  |  |  |  |  | X |  | **1** |
| 10.10 Reward (outcome) |  | X | X | X | X | X |  |  |  | **5** |
| 10.11 Future punishment | X |  |  |  |  |  | X |  |  | **2** |
| 11. Regulation |  |  |  |  |  |  |  |  |  |  |
| 11.1 Pharmacological support |  |  |  |  |  |  |  |  |  | **0** |
| 11.2 Reduce negative emotions |  | - | - | - | X | X |  |  | X | **3** |
| 11.3 Conserving mental resources |  | X | X | X | X | X |  |  |  | **5** |
| 11.4 Paradoxical instructions |  |  |  |  |  |  |  |  |  | **0** |
| 12. Antecedents |  |  |  |  |  |  |  |  |  |  |
| 12.1 Restructuring the physical environment | X | X | X | X | X | X | X | X | X | **9** |
| 12.2 Restructuring the social environment |  | X | X | X |  |  |  |  |  | **3** |
| 12.3 Avoidance/reducing exposure to cues for the behavior |  |  |  |  |  |  |  |  |  | **0** |
| 12.4 Distraction |  |  |  |  |  |  |  |  |  | **0** |
| 12.5 Adding objects to the environment | X | X | X | X | X | X | X | X | X | **9** |
| 12.6 Body changes |  |  |  |  |  |  |  |  |  | **0** |
| 13. Identity |  |  |  |  |  |  |  |  |  |  |
| 13.1 Identification of the self as role model |  |  |  |  |  |  |  |  |  | **0** |
| 13.2 Framing/reframing |  |  |  |  | X | X |  |  |  | **2** |
| 13.3 Incompatible beliefs |  |  |  |  |  |  |  |  |  | **0** |
| 13.4 Valued self-identity |  |  |  |  |  |  |  |  |  | **0** |
| 13.5 Identity associated with changed behavior |  |  |  |  |  |  |  |  |  | **0** |
| 14. Scheduled consequences |  |  |  |  |  |  |  |  |  |  |
| 14.1 Behavior cost |  |  |  |  |  |  |  |  |  | **0** |
| 14.2 Punishment |  |  |  |  |  |  |  |  |  | **0** |
| 14.3 Remove reward |  | X | X | X | X | X | X |  |  | **6** |
| 14.4 Reward approximation |  | X | X | X | X | X |  |  |  | **5** |
| 14.5 Rewarding completion |  | X | X | X | X | X | X |  | X | **7** |
| 14.6 Situation-specific reward |  | X | X | X |  |  |  |  |  | **3** |
| 14.7 Reward incompatible behavior |  |  |  |  |  |  |  |  |  | **0** |
| 14.8 Reward alternative behavior | X |  |  |  |  |  |  |  |  | **1** |
| 14.9 Reduce reward frequency |  | X | X | X |  |  |  |  |  | **3** |
| 14.10 Remove punishment |  |  |  |  |  |  |  |  |  | **0** |
| 15. Self-belief |  |  |  |  |  |  |  |  |  |  |
| 15.1 Verbal persuasion about capability |  |  |  |  |  |  |  |  |  | **0** |
| 15.2 Mental rehearsal of successful performance behavior |  |  |  |  | X | X |  |  |  | **2** |
| 15.3 Focus on past success | X | X | X | X | X | X | X | X |  | **8** |
| 15.4 Self-talk |  |  |  |  |  |  |  |  |  | **0** |
| 16. Covert learning |  |  |  |  |  |  |  |  |  |  |
| 16.1 Imaginary punishment |  |  |  |  |  |  |  |  |  | **0** |
| 16.2 Imaginary reward |  |  |  |  |  |  |  |  |  | **0** |
| 16.3 Vicarious consequences |  |  |  |  |  |  |  |  |  | **0** |
| **TOTAL** | **20** | **43** | **43** | **43** | **36** | **36** | **25** | **23** | **19** |  |

**(-)** The BCT is likely available as a premium feature

**Further specifications for coding of several techniques used by coders, Table 1:**

1.1 Goal setting (behavior): Daily and more distal goals were counted. If the only goal-setting was in small challenges (e.g., step as much as possible for one minute), it was not counted.

1.3 Goal setting (outcome): the outcome was weight.

1.4 Action planning: badges and challenges that encouraged certain behaviors and/or activity on particular dates/times of day were counted. The user is aware of these badges/challenges prior to the deadline which allows for action planning.

1.5 Review behavior goals: the ability to change activity goals was counted. Several apps also suggested that users review their goals.

1.8 Behavior contract: the user agreeing to a step or physical activity goal

1.9 Commitment: asking users to agree to a challenging, small goal was counted.

2.3 Self-monitoring of behavior: all automatic monitoring by the monitors was considered self-monitoring.

2.6 Biofeedback: hatched squares indicate that biofeedback is available within the app if an additional sensor is purchased (e.g., scale, heart rate monitor, blood pressure monitor).

3.1 Social support (unspecified): the ability to friend or follow other users, create teams or groups, or otherwise interact with others were counted.

3.2 Social support (practical): the ability to walk/exercise simultaneously with others or interact with others during an activity session was counted.

3.3 Social support (emotional): the ability to send and receive messages, emoticons, cheers, etc. was counted.

4.1 Instruction on how to perform the behavior: only instructions related to performance of exercise activities were counted.

6.2 Social comparison: competition and leaderboards that compare activity between “friends”

7.1 Prompts/cues: push notifications (notifications that cause smartphones to vibrate/chime and display a message) and monitor-based cues (vibrations, flashing lights, messages on the display) were counted.

8.7 Graded tasks: though theoretically any monitor/app that included user-entered goals could provide graded tasks, this technique was only coded as present when graded tasks were explicitly encouraged by the app. For example, different badges available for increasing levels of difficulty/effort were counted.

9.1 Credible source: links to credible websites (Centers for Disease Control, various University-based sites) were counted.

10.3 Non-specific reward: virtual rewards such as badges, trophies, and achievements were counted as non-specific rewards rather than imaginary rewards.

10.4 Social reward: ability to share progress/goal achievement to social networks was counted. If positive social interactions occurred within the app (for example, amongst members of a group or team), the interactions were coded as social support.

10.10 Reward (outcome): badges related to weight loss were counted.

12.5 Adding objects to the environment: activity monitors were considered additions to the environment.

14.6 Situation-specific reward: badges/trophies/achievements related to specific situations, such as reaching one’s daily goal on Valentine’s Day or achieving a certain step count within thirty minutes, were counted.

14.7 Reward incompatible behavior: badges for breaking up sedentary behavior bouts were counted.

15.3 Focus on past success: to be counted as this technique, rather than just as feedback, an app must have provided some kind of special emphasis on comparing past behavior to present behavior. Emails that provided an overview of weekly, monthly, and in some cases yearly activity and goal achievement were counted.
