# Supplementary material for: Analysis of the Behavioral Change and Utility Features of Electronic Activity Monitors
Source: Technologies (Basel). Author manuscript; Available in PMC 2025 Jan 28. (PMC11774501; doi:10.3390/technologies8040075)
Supplement: Additional File 2 [file NIHMS2016016-supplement-Additional_File_2.pdf]

WEARABLE DEVICE: Amazfit
Reviewer: Dr. Koyya, Grace, Maddison

	BCT present?	Comments		BCT present?	Comments
1. Goals and planning			10.5 Social incentive	☐	
1.1 Goal setting (behavior)	✓	steps per day	10.6 Non-specific incentive	☐	
1.2 Problem solving	☐		10.7 Self-incentive	☐	
1.3 Goal setting (outcome)	✓		10.8 Incentive (outcome)	☐	
1.4 Action planning	☐		10.9 Self-reward	☐	
1.5 Review behavior goal(s)	✓		10.10 Reward (outcome)	☐	
1.6 Discrepancy between current behavior and goal	✓		10.11 Future punishment	✓	Broken streak
1.7 Review outcome goal(s)	✓		11. Regulation		
1.8 Behavioral contract	✓	agree on selecting a goal	11.1 Pharmacological support	☐	
1.9 Commitment	☐		11.2 Reduce negative emotions	☐	
2. Feedback and monitoring			11.3 Conserving mental resources	☐	
2.1 Monitoring of behavior by others without feedback	☐	The watch display allows for constant feedback	11.4 Paradoxical instructions	☐	
2.2 Feedback on behavior	✓		12. Antecedents		
2.3 Self-monitoring of behavior	✓		12.1 Restructuring the physical environment	✓	
2.4 Self-monitoring of outcome(s) of behavior	✓		12.2 Restructuring the social environment	☐	
2.5 Monitoring of outcome(s) of behavior without feedback	☐		12.3 Avoidance/reducing exposure to cues for the behavior	☐	
2.6 Biofeedback	✓	Heart rate	12.4 Distraction	☐	
2.7 Feedback on outcome(s) of behavior	✓		12.5 Adding objects to the environment	✓	
3. Social support			12.6 Body changes	☐	
3.1. Social support (unspecified)	✓		13. Identity		
3.2. Social support (practical)	☐		13.1 Identification of the self as role model	☐	

WEARABLE DEVICE: Amazfit
Reviewer: Dr. Koyya, Grace, Maddison

3.3. Social support (emotional)	✓	Able to "nudge" friends
4. Shaping knowledge		
4.1 Instruction on how to perform the behavior	☐	
4.2 Information about antecedents	☐	
4.3 Re-attribution	☐	
4.4 Behavioral experiments	☐	
5. Natural consequences		
5.1 Information about health consequences	☐	
5.2 Salience of consequences	☐	
5.3 Information about social and environmental consequences	☐	
5.4 Monitoring of emotional consequences	☐	
5.5 Anticipated regret	☐	
5.6 Information about emotional consequences	☐	
6. Comparison of behavior		
6.1 Demonstration of the behavior	☐	
6.2 Social comparison	✓	friends steps and weight
6.3 Information about others' approval	☐	
7. Associations		
7.1 Prompts/cues	✓	sedentary alerts
7.2 Cue signaling reward	✓	
7.3 Reduce prompts/cues	☐	
7.4 Remove access to the reward	☐	

13.2 Framing/reframing	☐	
13.3 Incompatible beliefs	☐	
13.4 Valued self-identity	☐	
13.5 Identity associated with changed behavior	☐	
14. Scheduled consequences		
14.1 Behavior cost	☐	
14.2 Punishment	☐	
14.3 Remove reward	✓	Loss of activity streak
14.4 Reward approximation	☐	
14.5 Rewarding completion	✓	
14.6 Situation-specific reward	☐	
14.7 Reward incompatible behavior	☐	
14.8 Reward alternative behavior	☐	
14.9 Reduce reward frequency	☐	
14.10 Remove punishment	☐	
15. Self-belief		
15.1 Verbal persuasion about capability	☐	
15.2 Mental rehearsal of successful performance	☐	
15.3 Focus on past success	✓	
15.4 Self-talk	☐	
16. Covert learning	☐	
16.1 Imaginary punishment	☐	

WEARABLE DEVICE: Amazfit

Reviewer: Dr. Koyya, Grace, Maddison

7.5 Remove aversive stimulus	☐		16.2 Imaginary reward	☐	
7.6 Satiation	☐		16.3 Vicarious consequences	☐	
7.7 Exposure	☐		**Functionality**		
7.8 Associative learning	☐		Battery lasts <1 day	☐	
**8 Repetition and substitution**			Battery lasts 1-2 days	☐	
8.1 Behavioral practice/rehearsal	☐		Battery lasts 3-4 days	☐	
8.2 Behavior substitution	☐		Battery lasts 5-6 days	✓	While wearing it at night
8.3 Habit formation	✓		Battery last ≥7 days	☐	
8.4 Habit reversal	☐		Device pairs with a phone/tablet	✓	
8.5 Overcorrection	☐		Device pairs with a computer	☐	
8.6 Generalisation of target behavior	☐		Device syncs with phone/table notifications	✓	
8.7 Graded tasks	☐		Device face activity display	✓	
**9. Comparison of outcomes**			Mobile app activity display	✓	
9.1 Credible source	☐		Computer activity display	☐	
9.2 Pros and cons	☐		Wrist worn	✓	
9.3 Comparative imagining of future outcomes	☐		Other wear options	☐	
**10. Reward and threat**			**Behavior monitoring**		
10.1 Material incentive (behavior)	☐		Sleep	✓	
10.2 Material reward (behavior)	☐		Nutrition	✓	
10.3 Non-specific reward	✓	Streaks	Sedentary	☐	
10.4 Social reward	✓		Exercise (workout tracking)	✓	Walking, Running, Cycling

Physical activity behaviors tracked:

Steps per Day	✓	Minutes per Day (total)	✓	Exercise minutes per Day	✓	Energy expenditure (total)	✓	Exercise energy expenditure	✓
Sitting (idle) time	✓	Heart rate	✓	Other: GPS	✓	Other:	☐	Other:	☐

WEARABLE DEVICE: Apple Watch
Reviewer: Dr. Koyya

	BCT present?	Comments
1. Goals and planning		
1.1 Goal setting (behavior)	✓	Recommendation but can select own goal
1.2 Problem solving	☐	
1.3 Goal setting (outcome)	☐	
1.4 Action planning	☐	
1.5 Review behavior goal(s)	✓	
1.6 Discrepancy between current behavior and goal	✓	
1.7 Review outcome goal(s)	☐	
1.8 Behavioral contract	✓	Confirm new goal
1.9 Commitment	✓	
2. Feedback and monitoring		
2.1 Monitoring of behavior by others without feedback	☐	
2.2 Feedback on behavior	✓	
2.3 Self-monitoring of behavior	✓	
2.4 Self-monitoring of outcome(s) of behavior	☐	
2.5 Monitoring of outcome(s) of behavior without feedback	☐	
2.6 Biofeedback	✓	heart rate
2.7 Feedback on outcome(s) of behavior	☐	
3. Social support		
3.1. Social support (unspecified)	✓	leaderboard and competition
3.2. Social support (practical)	☐	

	BCT present?	Comments
10.5 Social incentive	☐	
10.6 Non-specific incentive	✓	
10.7 Self-incentive	☐	
10.8 Incentive (outcome)	✓	Badges
10.9 Self-reward	☐	
10.10 Reward (outcome)	☐	
10.11 Future punishment	✓	lose streak
11. Regulation		
11.1 Pharmacological support	☐	
11.2 Reduce negative emotions	☐	
11.3 Conserving mental resources	☐	
11.4 Paradoxical instructions	☐	
12. Antecedents		
12.1 Restructuring the physical environment	✓	
12.2 Restructuring the social environment	☐	
12.3 Avoidance/reducing exposure to cues for the behavior	☐	
12.4 Distraction	☐	
12.5 Adding objects to the environment	✓	
12.6 Body changes	☐	
13. Identity		
13.1 Identification of the self as role model	☐	

WEARABLE DEVICE: Apple Watch
Reviewer: Dr. Koyya

3.3. Social support (emotional)	☐		13.2 Framing/reframing	☐	
4. Shaping knowledge			13.3 Incompatible beliefs	☐	
4.1 Instruction on how to perform the behavior	☐		13.4 Valued self-identity	☐	
4.2 Information about antecedents	☐		13.5 Identity associated with changed behavior	☐	
4.3 Re-attribution	☐		14. Scheduled consequences		
4.4 Behavioral experiments	☐		14.1 Behavior cost	☐	
5. Natural consequences			14.2 Punishment	☐	
5.1 Information about health consequences	☐		14.3 Remove reward	☐	
5.2 Salience of consequences	☐		14.4 Reward approximation	☐	
5.3 Information about social and environmental consequences	☐		14.5 Rewarding completion	☐	
5.4 Monitoring of emotional consequences	☐		14.6 Situation-specific reward	☐	
5.5 Anticipated regret	☐		14.7 Reward incompatible behavior	☐	
5.6 Information about emotional consequences	☐		14.8 Reward alternative behavior	☑	Sedentary and workout rewards
6. Comparison of behavior			14.9 Reduce reward frequency	☐	
6.1 Demonstration of the behavior	☐		14.10 Remove punishment	☐	
6.2 Social comparison	☑		15. Self-belief		
6.3 Information about others' approval	☐		15.1 Verbal persuasion about capability	☐	
7. Associations			15.2 Mental rehearsal of successful performance	☐	
7.1 Prompts/cues	☑		15.3 Focus on past success	☑	
7.2 Cue signaling reward	☐		15.4 Self-talk	☐	
7.3 Reduce prompts/cues	☐		16. Covert learning	☐	
7.4 Remove access to the reward	☐		16.1 Imaginary punishment	☐	

WEARABLE DEVICE: Apple Watch
Reviewer: Dr. Koyya

7.5 Remove aversive stimulus	☐		16.2 Imaginary reward	☐	
7.6 Satiation	☐		16.3 Vicarious consequences	☐	
7.7 Exposure	☐		Functionality		
7.8 Associative learning	☐		Battery lasts <1 day	☐	
8 Repetition and substitution			Battery lasts 1-2 days	☐	
8.1 Behavioral practice/rehearsal	☐		Battery lasts 3-4 days	✓	
8.2 Behavior substitution	☐		Battery lasts 5-6 days	☐	
8.3 Habit formation	☐		Battery last ≥7 days	☐	
8.4 Habit reversal	☐		Device pairs with a phone/tablet	✓	iPhone only
8.5 Overcorrection	☐		Device pairs with a computer	☐	
8.6 Generalisation of target behavior	☐		Device syncs with phone/table notifications	✓	
8.7 Graded tasks	✓	graded goals	Device face activity display	✓	
9. Comparison of outcomes			Mobile app activity display	✓	
9.1 Credible source	☐		Computer activity display	☐	
9.2 Pros and cons	☐		Wrist worn	✓	
9.3 Comparative imagining of future outcomes	☐		Other wear options	☐	
10. Reward and threat			Behavior monitoring		
10.1 Material incentive (behavior)	☐		Sleep	☐	
10.2 Material reward (behavior)	☐		Nutrition	☐	
10.3 Non-specific reward	✓		Sedentary	✓	
10.4 Social reward	☐		Exercise (workout tracking)	✓	

Physical activity behaviors tracked:

Steps per Day	✓	Minutes per Day (total)	☐	Exercise minutes per Day	✓	Energy expenditure (total)	✓	Exercise energy expenditure	✓
Sitting (idle) time	✓	Heart rate	✓	Other: Distance	✓	Other:	☐	Other:	☐

WEARABLE DEVICE: Fitbit Charge 3
Reviewer: Dr. Koyya, Maddison, Grace

	BCT present?	Comments		BCT present?	Comments
1. Goals and planning			10.5 Social incentive	☐	
1.1 Goal setting (behavior)	☑	self-selected, all behavior	10.6 Non-specific incentive	☑	badges/trophies
1.2 Problem solving	☐	feature may be available on Premium upgrade	10.7 Self-incentive	☐	
1.3 Goal setting (outcome)	☑		10.8 Incentive (outcome)	☑	
1.4 Action planning	☐		10.9 Self-reward	☐	
1.5 Review behavior goal(s)	☑		10.10 Reward (outcome)	☑	
1.6 Discrepancy between current behavior and goal	☑		10.11 Future punishment	☐	
1.7 Review outcome goal(s)	☑		11. Regulation		
1.8 Behavioral contract	☑	Agree on selecting a goal	11.1 Pharmacological support	☐	
1.9 Commitment	☑		11.2 Reduce negative emotions	☐	premium feature
2. Feedback and monitoring			11.3 Conserving mental resources	☑	Wellness reports
2.1 Monitoring of behavior by others without feedback	☐	The watch display allows for constant feedback	11.4 Paradoxical instructions	☐	
2.2 Feedback on behavior	☑		12. Antecedents		
2.3 Self-monitoring of behavior	☑		12.1 Restructuring the physical environment	☑	
2.4 Self-monitoring of outcome(s) of behavior	☑		12.2 Restructuring the social environment	☑	Creation of virtual social groups
2.5 Monitoring of outcome(s) of behavior without feedback	☐		12.3 Avoidance/reducing exposure to cues for the behavior	☐	
2.6 Biofeedback	☑	Heart rate	12.4 Distraction	☐	
2.7 Feedback on outcome(s) of behavior	☑		12.5 Adding objects to the environment	☑	
3. Social support			12.6 Body changes	☐	
3.1. Social support (unspecified)	☑	Large Fitbit groups and personal friends/family	13. Identity		
3.2. Social support (practical)	☐		13.1 Identification of the self as role model	☐	

WEARABLE DEVICE: Fitbit Charge 3
Reviewer: Dr. Koyya, Maddison, Grace

3.3. Social support (emotional)	✓	cheer/taunt, comments
4. Shaping knowledge		
4.1 Instruction on how to perform the behavior	✓	Available through program discovery but more options with premium upgrade
4.2 Information about antecedents	✓	Available through program discovery
4.3 Re-attribution	☐	
4.4 Behavioral experiments	☐	
5. Natural consequences		
5.1 Information about health consequences	✓	Irregular health comments
5.2 Salience of consequences	☐	
5.3 Information about social and environmental consequences	☐	
5.4 Monitoring of emotional consequences	☐	
5.5 Anticipated regret	✓	
5.6 Information about emotional consequences	✓	
6. Comparison of behavior		
6.1 Demonstration of the behavior	✓	Articles of people who were successful
6.2 Social comparison	✓	Leaderboard
6.3 Information about others' approval	✓	
7. Associations		
7.1 Prompts/cues	✓	sedentary alerts to take steps
7.2 Cue signaling reward	✓	
7.3 Reduce prompts/cues	☐	
7.4 Remove access to the reward	☐	

13.2 Framing/reframing	☐	
13.3 Incompatible beliefs	☐	
13.4 Valued self-identity	☐	
13.5 Identity associated with changed behavior	☐	
14. Scheduled consequences		
14.1 Behavior cost	☐	
14.2 Punishment	☐	
14.3 Remove reward	✓	Loss of activity streak
14.4 Reward approximation	✓	
14.5 Rewarding completion	✓	
14.6 Situation-specific reward	✓	
14.7 Reward incompatible behavior	☐	
14.8 Reward alternative behavior	☐	
14.9 Reduce reward frequency	✓	
14.10 Remove punishment	☐	
15. Self-belief		
15.1 Verbal persuasion about capability	☐	
15.2 Mental rehearsal of successful performance	☐	
15.3 Focus on past success	✓	
15.4 Self-talk	☐	
16. Covert learning	☐	
16.1 Imaginary punishment	☐	

WEARABLE DEVICE: Fitbit Charge 3
Reviewer: Dr. Koyya, Maddison, Grac[illegible]

7.5 Remove aversive stimulus	☐		16.2 Imaginary reward	☐	
7.6 Satiation	☐		16.3 Vicarious consequences	☐	
7.7 Exposure	☐		**Functionality**		
7.8 Associative learning	☐		Battery lasts <1 day	☐	
**8 Repetition and substitution**			Battery lasts 1-2 days	☐	
8.1 Behavioral practice/rehearsal	☐		Battery lasts 3-4 days	✓	
8.2 Behavior substitution	✓	Steps instead of sedentary time	Battery lasts 5-6 days	☐	
8.3 Habit formation	✓		Battery last ≥7 days	✓	While wearing it at night
8.4 Habit reversal	☐		Device pairs with a phone/tablet	✓	
8.5 Overcorrection	☐		Device pairs with a computer	☐	
8.6 Generalisation of target behavior	☐		Device syncs with phone/table notifications	✓	
8.7 Graded tasks	✓		Device face activity display	✓	
**9. Comparison of outcomes**			Mobile app activity display	✓	
9.1 Credible source	✓	In related blog articles	Computer activity display	☐	
9.2 Pros and cons	☐		Wrist worn	✓	
9.3 Comparative imagining of future outcomes	☐		Other wear options	☐	
**10. Reward and threat**			**Behavior monitoring**		
10.1 Material incentive (behavior)	☐		Sleep	✓	
10.2 Material reward (behavior)	☐		Nutrition	✓	
10.3 Non-specific reward	✓	Virtual badges/trophies	Sedentary	✓	
10.4 Social reward	✓	Likes/comments	Exercise (workout tracking)	✓	Most exercise types

Physical activity behaviors tracked:

Steps per Day	✓	Minutes per Day (total)	✓	Exercise minutes per Day	✓	Energy expenditure (total)	✓	Exercise energy expenditure	✓
Sitting (idle) time	✓	Heart rate	✓	Other: Female health	✓	Other: Distance	✓	Other: Floors	✓

WEARABLE DEVICE: Fitbit Ionic (Adidas)
Reviewer: Dr. Koyya, Maddison, Grace

	BCT present?	Comments		BCT present?	Comments
1. Goals and planning			10.5 Social incentive	☐	
1.1 Goal setting (behavior)	✓	self-selected, all behavior	10.6 Non-specific incentive	✓	badges/trohies
1.2 Problem solving	☐	feature may be available on Premium upgrade	10.7 Self-incentive	☐	
1.3 Goal setting (outcome)	✓		10.8 Incentive (outcome)	✓	
1.4 Action planning	☐		10.9 Self-reward	☐	
1.5 Review behavior goal(s)	✓		10.10 Reward (outcome)	✓	
1.6 Discrepancy between current behavior and goal	✓		10.11 Future punishment	☐	
1.7 Review outcome goal(s)	✓		11. Regulation		
1.8 Behavioral contract	✓	Agree on selecting a goal	11.1 Pharmacological support	☐	
1.9 Commitment	✓		11.2 Reduce negative emotions	☐	premium feature
2. Feedback and monitoring			11.3 Conserving mental resources	☐	
2.1 Monitoring of behavior by others without feedback	☐	the watch display allows for constant feedback	11.4 Paradoxical instructions	☐	
2.2 Feedback on behavior	✓		12. Antecedents		
2.3 Self-monitoring of behavior	✓		12.1 Restructuring the physical environment	✓	
2.4 Self-monitoring of outcome(s) of behavior	✓		12.2 Restructuring the social environment	✓	creation of virtual social groups
2.5 Monitoring of outcome(s) of behavior without feedback	☐		12.3 Avoidance/reducing exposure to cues for the behavior	☐	
2.6 Biofeedback	✓	heart rate	12.4 Distraction	☐	
2.7 Feedback on outcome(s) of behavior	✓		12.5 Adding objects to the environment	✓	
3. Social support			12.6 Body changes	☐	
3.1. Social support (unspecified)	✓	Large fitbit groups and personal friends/family	13. Identity		
3.2. Social support (practical)	☐		13.1 Identification of the self as role model	☐	

WEARABLE DEVICE: Fitbit Ionic (Adidas)

Reviewer: Dr. Koyya, Maddison, Grace

3.3. Social support (emotional)	✓	cheer/taunt, comments	13.2 Framing/reframing	☐	
4. Shaping knowledge			13.3 Incompatible beliefs	☐	
4.1 Instruction on how to perform the behavior	✓	Available through program discovery	13.4 Valued self-identity	☐	
4.2 Information about antecedents	✓	Available through program discoery	13.5 Identity associated with changed behavior	☐	
4.3 Re-attribution	☐		14. Scheduled consequences		
4.4 Behavioral experiments	☐		14.1 Behavior cost	☐	
5. Natural consequences			14.2 Punishment	☐	
5.1 Information about health consequences	✓	irregular health comments	14.3 Remove reward	✓	Loss of activity streak
5.2 Salience of consequences	☐		14.4 Reward approximation	✓	
5.3 Information about social and environmental consequences	☐		14.5 Rewarding completion	✓	
5.4 Monitoring of emotional consequences	☐		14.6 Situation-specific reward	✓	
5.5 Anticipated regret	✓		14.7 Reward incompatible behavior	☐	
5.6 Information about emotional consequences	✓		14.8 Reward alternative behavior	✓	
6. Comparison of behavior			14.9 Reduce reward frequency	☐	
6.1 Demonstration of the behavior	✓	Articles of people who were successful	14.10 Remove punishment	☐	
6.2 Social comparison	✓	Leaderboard	15. Self-belief		
6.3 Information about others' approval	✓		15.1 Verbal persuasion about capability	☐	
7. Associations			15.2 Mental rehearsal of successful performance	☐	
7.1 Prompts/cues	✓	sedentary alerts to take steps	15.3 Focus on past success	✓	
7.2 Cue signaling reward	✓		15.4 Self-talk	☐	
7.3 Reduce prompts/cues	☐		16. Covert learning	☐	
7.4 Remove access to the reward	☐		16.1 Imaginary punishment	☐	

WEARABLE DEVICE: Fitbit Ionic (Adidas)
Reviewer: Dr. Koyya, Maddison, Grac

7.5 Remove aversive stimulus			16.2 Imaginary reward		
7.6 Satiation			16.3 Vicarious consequences		
7.7 Exposure			Functionality		
7.8 Associative learning			Battery lasts <1 day		
8 Repetition and substitution			Battery lasts 1-2 days		
8.1 Behavioral practice/rehearsal		Premium feature	Battery lasts 3-4 days		
8.2 Behavior substitution	✓	steps instead of sedentary time	Battery lasts 5-6 days	✓	
8.3 Habit formation	✓		Battery last ≥7 days	✓	While wearing it to sleep
8.4 Habit reversal			Device pairs with a phone/tablet	✓	
8.5 Overcorrection			Device pairs with a computer		
8.6 Generalisation of target behavior			Device syncs with phone/table notifications	✓	
8.7 Graded tasks	✓		Device face activity display	✓	
9. Comparison of outcomes			Mobile app activity display	✓	
9.1 Credible source	✓	In related blog articles	Computer activity display		
9.2 Pros and cons			Wrist worn	✓	
9.3 Comparative imagining of future outcomes			Other wear options		
10. Reward and threat			Behavior monitoring		
10.1 Material incentive (behavior)			Sleep	✓	
10.2 Material reward (behavior)			Nutrition	✓	
10.3 Non-specific reward	✓	Virtual badges/trophies	Sedentary	✓	
10.4 Social reward	✓	Likes/comments	Exercise (workout tracking)	✓	Most exercise types

Physical activity behaviors tracked:

Steps per Day	✓	Minutes per Day (total)		Exercise minutes per Day	✓	Energy expenditure (total)	✓	Exercise energy expenditure	✓
Sitting (idle) time	✓	Heart rate	✓	Other: Female health	✓	Other: Distance	✓	Other: Floors	✓

WEARABLE DEVICE: Fitbit Versa 2
Reviewer: Dr. Koyya, Grace, Maddison

	BCT present?	Comments		BCT present?	Comments
1. Goals and planning			10.5 Social incentive	☐	
1.1 Goal setting (behavior)	☑	self-selected, all behavior	10.6 Non-specific incentive	☑	badges/trophies
1.2 Problem solving	☐	Feature may be available on premium upgrade	10.7 Self-incentive	☐	
1.3 Goal setting (outcome)	☑		10.8 Incentive (outcome)	☑	
1.4 Action planning	☐		10.9 Self-reward	☐	
1.5 Review behavior goal(s)	☑		10.10 Reward (outcome)	☑	
1.6 Discrepancy between current behavior and goal	☑		10.11 Future punishment	☐	
1.7 Review outcome goal(s)	☑		11. Regulation		
1.8 Behavioral contract	☑	Agree on selecting goal	11.1 Pharmacological support	☐	
1.9 Commitment	☑		11.2 Reduce negative emotions	☐	premium feature
2. Feedback and monitoring			11.3 Conserving mental resources	☑	Intro to healthy habits guided programs
2.1 Monitoring of behavior by others without feedback	☐	the watch allows for constant feedback	11.4 Paradoxical instructions	☐	
2.2 Feedback on behavior	☑		12. Antecedents		
2.3 Self-monitoring of behavior	☑		12.1 Restructuring the physical environment	☑	
2.4 Self-monitoring of outcome(s) of behavior	☑		12.2 Restructuring the social environment	☑	Creation of virtual social groups
2.5 Monitoring of outcome(s) of behavior without feedback	☐		12.3 Avoidance/reducing exposure to cues for the behavior	☐	
2.6 Biofeedback	☑	heart rate	12.4 Distraction	☐	
2.7 Feedback on outcome(s) of behavior	☑		12.5 Adding objects to the environment	☑	
3. Social support			12.6 Body changes	☐	
3.1. Social support (unspecified)	☑	Large Fitbit groups and personal friends/family	13. Identity		
3.2. Social support (practical)	☐		13.1 Identification of the self as role model	☐	

WEARABLE DEVICE: Fitbit Versa 2
Reviewer: Dr. Koyya, Grace, Maddisor

3.3. Social support (emotional)	☑	
4. Shaping knowledge		
4.1 Instruction on how to perform the behavior	☑	
4.2 Information about antecedents	☑	
4.3 Re-attribution	☐	
4.4 Behavioral experiments	☐	
5. Natural consequences		
5.1 Information about health consequences	☑	
5.2 Salience of consequences	☐	
5.3 Information about social and environmental consequences	☐	
5.4 Monitoring of emotional consequences	☐	
5.5 Anticipated regret	☑	
5.6 Information about emotional consequences	☑	
6. Comparison of behavior		
6.1 Demonstration of the behavior	☑	Articles of people who were successful
6.2 Social comparison	☑	
6.3 Information about others' approval	☑	
7. Associations		
7.1 Prompts/cues	☑	
7.2 Cue signaling reward	☑	
7.3 Reduce prompts/cues	☐	
7.4 Remove access to the reward	☐	

13.2 Framing/reframing	☐	
13.3 Incompatible beliefs	☐	
13.4 Valued self-identity	☐	
13.5 Identity associated with changed behavior	☐	
14. Scheduled consequences		
14.1 Behavior cost	☐	
14.2 Punishment	☐	
14.3 Remove reward	☑	Loss of activity streak
14.4 Reward approximation	☑	
14.5 Rewarding completion	☑	
14.6 Situation-specific reward	☑	
14.7 Reward incompatible behavior	☐	
14.8 Reward alternative behavior	☐	
14.9 Reduce reward frequency	☑	
14.10 Remove punishment	☐	
15. Self-belief		
15.1 Verbal persuasion about capability	☐	
15.2 Mental rehearsal of successful performance	☐	
15.3 Focus on past success	☑	
15.4 Self-talk	☐	
16. Covert learning	☐	
16.1 Imaginary punishment	☐	

WEARABLE DEVICE: Fitbit Versa 2
Reviewer: Dr. Koyya, Grace, Maddison

7.5 Remove aversive stimulus	☐		16.2 Imaginary reward	☐	
7.6 Satiation	☐		16.3 Vicarious consequences	☐	
7.7 Exposure	☐		Functionality		
7.8 Associative learning	☐		Battery lasts <1 day	☐	
8 Repetition and substitution			Battery lasts 1-2 days	☐	
8.1 Behavioral practice/rehearsal	☐		Battery lasts 3-4 days	☐	
8.2 Behavior substitution	✓	Steps instead of sedentary time	Battery lasts 5-6 days	✓	
8.3 Habit formation	✓		Battery last ≥7 days	✓	While wearing it at night
8.4 Habit reversal	☐		Device pairs with a phone/tablet	✓	
8.5 Overcorrection	☐		Device pairs with a computer	☐	
8.6 Generalisation of target behavior	☐		Device syncs with phone/table notifications	✓	
8.7 Graded tasks	✓		Device face activity display	✓	
9. Comparison of outcomes			Mobile app activity display	✓	
9.1 Credible source	✓		Computer activity display	☐	
9.2 Pros and cons	☐		Wrist worn	✓	
9.3 Comparative imagining of future outcomes	☐		Other wear options	☐	
10. Reward and threat			Behavior monitoring		
10.1 Material incentive (behavior)	☐		Sleep	✓	
10.2 Material reward (behavior)	☐		Nutrition	✓	
10.3 Non-specific reward	✓		Sedentary	✓	
10.4 Social reward	✓		Exercise (workout tracking)	✓	Most exercise types

Physical activity behaviors tracked:

Steps per Day	✓	Minutes per Day (total)	✓	Exercise minutes per Day	✓	Energy expenditure (total)	✓	Exercise energy expenditure	✓
Sitting (idle) time	✓	Heart rate	✓	Other: Female health	✓	Other: Distance	✓	Other: Floors	✓

WEARABLE DEVICE: Samsung galaxy watch
Reviewer: Dr. Koyya, Maddison, Grace

	BCT present?	Comments		BCT present?	Comments
1. Goals and planning			10.5 Social incentive	✓	With other Samsung apps
1.1 Goal setting (behavior)	✓	custom calorie burn, minutes, move hours	10.6 Non-specific incentive	☐	
1.2 Problem solving	☐		10.7 Self-incentive	☐	
1.3 Goal setting (outcome)	✓		10.8 Incentive (outcome)	☐	
1.4 Action planning	☐		10.9 Self-reward	✓	
1.5 Review behavior goal(s)	✓	Weekly review	10.10 Reward (outcome)	☐	
1.6 Discrepancy between current behavior and goal	✓		10.11 Future punishment	☐	
1.7 Review outcome goal(s)	✓		11. Regulation		
1.8 Behavioral contract	✓		11.1 Pharmacological support	☐	
1.9 Commitment	✓		11.2 Reduce negative emotions	☐	
2. Feedback and monitoring			11.3 Conserving mental resources	☐	
2.1 Monitoring of behavior by others without feedback	☐		11.4 Paradoxical instructions	☐	
2.2 Feedback on behavior	✓		12. Antecedents		
2.3 Self-monitoring of behavior	✓		12.1 Restructuring the physical environment	✓	
2.4 Self-monitoring of outcome(s) of behavior	☐		12.2 Restructuring the social environment	☐	
2.5 Monitoring of outcome(s) of behavior without feedback	☐		12.3 Avoidance/reducing exposure to cues for the behavior	☐	
2.6 Biofeedback	✓	heart rate	12.4 Distraction	☐	
2.7 Feedback on outcome(s) of behavior	☐		12.5 Adding objects to the environment	✓	
3. Social support			12.6 Body changes	☐	
3.1. Social support (unspecified)	✓		13. Identity		
3.2. Social support (practical)	☐		13.1 Identification of the self as role model	☐	

WEARABLE DEVICE: Samsung galaxy watch
Reviewer: Dr. Koyya, Maddison, Grac

3.3. Social support (emotional)	☐		13.2 Framing/reframing	☐	
4. Shaping knowledge			13.3 Incompatible beliefs	☐	
4.1 Instruction on how to perform the behavior	✓		13.4 Valued self-identity	☐	
4.2 Information about antecedents	☐		13.5 Identity associated with changed behavior	☐	
4.3 Re-attribution	☐		14. Scheduled consequences		
4.4 Behavioral experiments	☐		14.1 Behavior cost	☐	
5. Natural consequences			14.2 Punishment	☐	
5.1 Information about health consequences	☐		14.3 Remove reward	☐	
5.2 Salience of consequences	☐		14.4 Reward approximation	☐	
5.3 Information about social and environmental consequences	☐		14.5 Rewarding completion	☐	
5.4 Monitoring of emotional consequences	☐		14.6 Situation-specific reward	☐	
5.5 Anticipated regret	☐		14.7 Reward incompatible behavior	☐	
5.6 Information about emotional consequences	☐		14.8 Reward alternative behavior	☐	
6. Comparison of behavior			14.9 Reduce reward frequency	☐	
6.1 Demonstration of the behavior	☐		14.10 Remove punishment	☐	
6.2 Social comparison	✓		15. Self-belief		
6.3 Information about others' approval	☐		15.1 Verbal persuasion about capability	☐	
7. Associations			15.2 Mental rehearsal of successful performance	☐	
7.1 Prompts/cues	✓	Sedentary alerts	15.3 Focus on past success	✓	Comparison of current and past performance
7.2 Cue signaling reward	☐		15.4 Self-talk	☐	
7.3 Reduce prompts/cues	☐		16. Covert learning	☐	
7.4 Remove access to the reward	☐		16.1 Imaginary punishment	☐	

WEARABLE DEVICE: Samsung galaxy watch
Reviewer: Dr. Koyya, Maddison, Grac[illegible]

7.5 Remove aversive stimulus	☐		16.2 Imaginary reward	☐	
7.6 Satiation	☐		16.3 Vicarious consequences	☐	
7.7 Exposure	☐		Functionality		
7.8 Associative learning	☐		Battery lasts <1 day	☐	
8 Repetition and substitution			Battery lasts 1-2 days	☑	When using "Wifi always on"
8.1 Behavioral practice/rehearsal	☑		Battery lasts 3-4 days	☑	Without "wifi always on"
8.2 Behavior substitution	☑	Activity for sedentary behavior	Battery lasts 5-6 days	☐	
8.3 Habit formation	☑		Battery last ≥7 days	☐	
8.4 Habit reversal	☐		Device pairs with a phone/tablet	☑	Android phone only
8.5 Overcorrection	☐		Device pairs with a computer	☐	
8.6 Generalisation of target behavior	☐		Device syncs with phone/table notifications	☑	Galaxy user
8.7 Graded tasks	☐		Device face activity display	☑	iPhone user
9. Comparison of outcomes			Mobile app activity display	☐	
9.1 Credible source	☐		Computer activity display	☐	
9.2 Pros and cons	☐		Wrist worn	☑	
9.3 Comparative imagining of future outcomes	☐		Other wear options	☐	
10. Reward and threat			Behavior monitoring		
10.1 Material incentive (behavior)	☐		Sleep	☑	
10.2 Material reward (behavior)	☐		Nutrition	☑	
10.3 Non-specific reward	☑		Sedentary	☑	
10.4 Social reward	☐		Exercise (workout tracking)	☑	

Physical activity behaviors tracked:

Steps per Day	☑	Minutes per Day (total)	☐	Exercise minutes per Day	☑	Energy expenditure (total)	☐	Exercise energy expenditure	☑
Sitting (idle) time	☑	Heart rate	☑	Other: Stress	☑	Other:	☐	Other:	☐

WEARABLE DEVICE: Vivomove HR
Reviewer: Dr. Koyya, Maddison

	BCT present?	Comments		BCT present?	Comments
1. Goals and planning			10.5 Social incentive	☐	
1.1 Goal setting (behavior)	☑	Auto or custom	10.6 Non-specific incentive	☑	
1.2 Problem solving	☐		10.7 Self-incentive	☐	
1.3 Goal setting (outcome)	☑		10.8 Incentive (outcome)	☐	
1.4 Action planning	☐		10.9 Self-reward	☐	
1.5 Review behavior goal(s)	☑		10.10 Reward (outcome)	☑	
1.6 Discrepancy between current behavior and goal	☑		10.11 Future punishment	☐	
1.7 Review outcome goal(s)	☑		11. Regulation		
1.8 Behavioral contract	☐		11.1 Pharmacological support	☐	
1.9 Commitment	☑		11.2 Reduce negative emotions	☑	rates stress levels
2. Feedback and monitoring			11.3 Conserving mental resources	☑	
2.1 Monitoring of behavior by others without feedback	☐		11.4 Paradoxical instructions	☐	
2.2 Feedback on behavior	☑		12. Antecedents		
2.3 Self-monitoring of behavior	☑		12.1 Restructuring the physical environment	☑	
2.4 Self-monitoring of outcome(s) of behavior	☑		12.2 Restructuring the social environment	☐	
2.5 Monitoring of outcome(s) of behavior without feedback	☑		12.3 Avoidance/reducing exposure to cues for the behavior	☐	
2.6 Biofeedback	☑	heart rate	12.4 Distraction	☐	
2.7 Feedback on outcome(s) of behavior	☐		12.5 Adding objects to the environment	☑	
3. Social support			12.6 Body changes	☐	
3.1. Social support (unspecified)	☑	see connection's activity	13. Identity		
3.2. Social support (practical)	☐		13.1 Identification of the self as role model	☐	

WEARABLE DEVICE: Vivomove HR
Reviewer: Dr. Koyya, Maddison

3.3. Social support (emotional)	✓	
4. Shaping knowledge		
4.1 Instruction on how to perform the behavior	✓	
4.2 Information about antecedents	✓	
4.3 Re-attribution	☐	
4.4 Behavioral experiments	✓	
5. Natural consequences		
5.1 Information about health consequences	✓	
5.2 Salience of consequences	☐	
5.3 Information about social and environmental consequences	☐	
5.4 Monitoring of emotional consequences	☐	
5.5 Anticipated regret	☐	
5.6 Information about emotional consequences	☐	
6. Comparison of behavior		
6.1 Demonstration of the behavior	☐	
6.2 Social comparison	✓	Insight comparison to people of same age/sex
6.3 Information about others' approval	☐	
7. Associations		
7.1 Prompts/cues	✓	sedentary alerts to take steps
7.2 Cue signaling reward	✓	
7.3 Reduce prompts/cues	✓	
7.4 Remove access to the reward	☐	

13.2 Framing/reframing	✓	
13.3 Incompatible beliefs	☐	
13.4 Valued self-identity	☐	
13.5 Identity associated with changed behavior	☐	
14. Scheduled consequences		
14.1 Behavior cost	☐	
14.2 Punishment	☐	
14.3 Remove reward	✓	
14.4 Reward approximation	✓	
14.5 Rewarding completion	✓	
14.6 Situation-specific reward	☐	
14.7 Reward incompatible behavior	☐	
14.8 Reward alternative behavior	☐	
14.9 Reduce reward frequency	☐	
14.10 Remove punishment	☐	
15. Self-belief		
15.1 Verbal persuasion about capability	☐	
15.2 Mental rehearsal of successful performance	✓	
15.3 Focus on past success	✓	
15.4 Self-talk	☐	
16. Covert learning	☐	
16.1 Imaginary punishment	☐	

WEARABLE DEVICE: Vivomove HR

Reviewer: Dr. Koyya, Maddison

7.5 Remove aversive stimulus	☐		16.2 Imaginary reward	☐	
7.6 Satiation	☐		16.3 Vicarious consequences	☐	
7.7 Exposure	☐		Functionality		
7.8 Associative learning	☐		Battery lasts <1 day	☐	
8 Repetition and substitution			Battery lasts 1-2 days	☐	
8.1 Behavioral practice/rehearsal	☐		Battery lasts 3-4 days	✓	
8.2 Behavior substitution	☐		Battery lasts 5-6 days	✓	While wearing it to bed
8.3 Habit formation	✓		Battery last ≥7 days	☐	
8.4 Habit reversal	✓		Device pairs with a phone/tablet	✓	
8.5 Overcorrection	☐		Device pairs with a computer	☐	
8.6 Generalisation of target behavior	☐		Device syncs with phone/table notifications	✓	
8.7 Graded tasks	✓		Device face activity display	✓	
9. Comparison of outcomes			Mobile app activity display	✓	
9.1 Credible source	☐		Computer activity display	☐	
9.2 Pros and cons	☐		Wrist worn	✓	
9.3 Comparative imagining of future outcomes	✓		Other wear options	☐	
10. Reward and threat			Behavior monitoring		
10.1 Material incentive (behavior)	☐		Sleep	✓	
10.2 Material reward (behavior)	☐		Nutrition	☐	Hydration, partners with MyFitness Pal
10.3 Non-specific reward	✓		Sedentary	✓	
10.4 Social reward	☐		Exercise (workout tracking)	✓	

Physical activity behaviors tracked:

Steps per Day	✓	Minutes per Day (total)	✓	Exercise minutes per Day	✓	Energy expenditure (total)	✓	Exercise energy expenditure	✓
Sitting (idle) time	☐	Heart rate	✓	Other: Menstrual tracking	✓	Other: Stress	✓	Other: Floors	✓

WEARABLE DEVICE: Garmin Vivosmart 4
Reviewer: Dr. Koyya, Maddison, Grace

	BCT present?	Comments		BCT present?	Comments
1. Goals and planning			10.5 Social incentive	☐	
1.1 Goal setting (behavior)	✓	Auto or custom	10.6 Non-specific incentive	✓	
1.2 Problem solving	☐		10.7 Self-incentive	☐	
1.3 Goal setting (outcome)	✓		10.8 Incentive (outcome)	☐	
1.4 Action planning	☐		10.9 Self-reward	☐	
1.5 Review behavior goal(s)	✓		10.10 Reward (outcome)	✓	
1.6 Discrepancy between current behavior and goal	✓		10.11 Future punishment	☐	
1.7 Review outcome goal(s)	☐		11. Regulation		
1.8 Behavioral contract	☐		11.1 Pharmacological support	☐	
1.9 Commitment	✓		11.2 Reduce negative emotions	✓	stress details
2. Feedback and monitoring			11.3 Conserving mental resources	✓	relax reminders-- guided breathing
2.1 Monitoring of behavior by others without feedback	☐		11.4 Paradoxical instructions	☐	
2.2 Feedback on behavior	✓		12. Antecedents		
2.3 Self-monitoring of behavior	✓		12.1 Restructuring the physical environment	✓	
2.4 Self-monitoring of outcome(s) of behavior	✓		12.2 Restructuring the social environment	☐	
2.5 Monitoring of outcome(s) of behavior without feedback	✓		12.3 Avoidance/reducing exposure to cues for the behavior	☐	
2.6 Biofeedback	✓	heart rate and oxygen	12.4 Distraction	☐	
2.7 Feedback on outcome(s) of behavior	☐		12.5 Adding objects to the environment	✓	
3. Social support			12.6 Body changes	☐	
3.1. Social support (unspecified)	✓	see connection's activity	13. Identity		
3.2. Social support (practical)	☐		13.1 Identification of the self as role model	☐	

WEARABLE DEVICE: Garmin Vivosmart 4
Reviewer: Dr. Koyya, Maddison, Grac

3.3. Social support (emotional)	✓	
4. Shaping knowledge		
4.1 Instruction on how to perform the behavior	✓	Find workouts
4.2 Information about antecedents	✓	
4.3 Re-attribution	☐	
4.4 Behavioral experiments	✓	
5. Natural consequences		
5.1 Information about health consequences	✓	
5.2 Salience of consequences	☐	
5.3 Information about social and environmental consequences	☐	
5.4 Monitoring of emotional consequences	☐	
5.5 Anticipated regret	☐	
5.6 Information about emotional consequences	☐	
6. Comparison of behavior		
6.1 Demonstration of the behavior	☐	
6.2 Social comparison	✓	Insight comparison to people of same age/sex
6.3 Information about others' approval	☐	
7. Associations		
7.1 Prompts/cues	✓	
7.2 Cue signaling reward	✓	
7.3 Reduce prompts/cues	☐	
7.4 Remove access to the reward	☐	

13.2 Framing/reframing	✓	
13.3 Incompatible beliefs	☐	
13.4 Valued self-identity	☐	
13.5 Identity associated with changed behavior	☐	
14. Scheduled consequences		
14.1 Behavior cost	☐	
14.2 Punishment	☐	
14.3 Remove reward	✓	
14.4 Reward approximation	✓	
14.5 Rewarding completion	✓	
14.6 Situation-specific reward	☐	
14.7 Reward incompatible behavior	☐	
14.8 Reward alternative behavior	☐	
14.9 Reduce reward frequency	☐	
14.10 Remove punishment	☐	
15. Self-belief		
15.1 Verbal persuasion about capability	☐	
15.2 Mental rehearsal of successful performance	✓	
15.3 Focus on past success	✓	
15.4 Self-talk	☐	
16. Covert learning	☐	
16.1 Imaginary punishment	☐	

WEARABLE DEVICE: Garmin Vivosmart 4
Reviewer: Dr. Koyya, Maddison, Grace

7.5 Remove aversive stimulus	☐		16.2 Imaginary reward	☐	
7.6 Satiation	☐		16.3 Vicarious consequences	☐	
7.7 Exposure	☐		Functionality		
7.8 Associative learning	☐		Battery lasts <1 day	☐	
8 Repetition and substitution			Battery lasts 1-2 days	☐	
8.1 Behavioral practice/rehearsal	☐		Battery lasts 3-4 days	✓	
8.2 Behavior substitution	☐		Battery lasts 5-6 days	☐	
8.3 Habit formation	✓		Battery last ≥7 days	☐	
8.4 Habit reversal	✓		Device pairs with a phone/tablet	✓	
8.5 Overcorrection	☐		Device pairs with a computer	☐	
8.6 Generalisation of target behavior	☐		Device syncs with phone/table notifications	✓	
8.7 Graded tasks	✓		Device face activity display	✓	
9. Comparison of outcomes			Mobile app activity display	✓	
9.1 Credible source	☐		Computer activity display	☐	
9.2 Pros and cons	☐		Wrist worn	✓	
9.3 Comparative imagining of future outcomes	✓		Other wear options	☐	
10. Reward and threat			Behavior monitoring		
10.1 Material incentive (behavior)	☐		Sleep	✓	
10.2 Material reward (behavior)	☐		Nutrition	☐	Partners with MyFitness Pal
10.3 Non-specific reward	✓		Sedentary	✓	
10.4 Social reward	☐		Exercise (workout tracking)	✓	Auto-detect activities

Physical activity behaviors tracked:

Steps per Day	✓	Minutes per Day (total)	✓	Exercise minutes per Day	✓	Energy expenditure (total)	✓	Exercise energy expenditure	✓
Sitting (idle) time	☐	Heart rate	✓	Other: Stress; menstrual cycle	✓	Other: Hydration	✓	Other: Floors	✓

WEARABLE DEVICE: Withings Steel Active
Reviewer: Dr. Koyya, Maddison, Grace

	BCT present?	Comments
1. Goals and planning		
1.1 Goal setting (behavior)	✓	Self-selected
1.2 Problem solving		
1.3 Goal setting (outcome)		
1.4 Action planning		
1.5 Review behavior goal(s)	✓	
1.6 Discrepancy between current behavior and goal	✓	
1.7 Review outcome goal(s)		
1.8 Behavioral contract	✓	Agreeing on a step goal
1.9 Commitment	✓	
2. Feedback and monitoring		
2.1 Monitoring of behavior by others without feedback		
2.2 Feedback on behavior	✓	
2.3 Self-monitoring of behavior	✓	
2.4 Self-monitoring of outcome(s) of behavior		
2.5 Monitoring of outcome(s) of behavior without feedback		
2.6 Biofeedback	✓	heart rate
2.7 Feedback on outcome(s) of behavior		
3. Social support		
3.1. Social support (unspecified)	✓	Leader-board competition with friends/family
3.2. Social support (practical)		

	BCT present?	Comments
10.5 Social incentive		
10.6 Non-specific incentive	✓	
10.7 Self-incentive		
10.8 Incentive (outcome)		
10.9 Self-reward		
10.10 Reward (outcome)		
10.11 Future punishment		
11. Regulation		
11.1 Pharmacological support		
11.2 Reduce negative emotions	✓	Meditation option
11.3 Conserving mental resources		
11.4 Paradoxical instructions		
12. Antecedents		
12.1 Restructuring the physical environment	✓	
12.2 Restructuring the social environment		
12.3 Avoidance/reducing exposure to cues for the behavior		
12.4 Distraction		
12.5 Adding objects to the environment	✓	
12.6 Body changes		
13. Identity		
13.1 Identification of the self as role model		

WEARABLE DEVICE: Withings Steel Active
Reviewer: Dr. Koyya, Maddison, Grac[illegible]

3.3. Social support (emotional)	☐		13.2 Framing/reframing	☐	
4. Shaping knowledge			13.3 Incompatible beliefs	☐	
4.1 Instruction on how to perform the behavior	☑		13.4 Valued self-identity	☐	
4.2 Information about antecedents	☐		13.5 Identity associated with changed behavior	☐	
4.3 Re-attribution	☐		14. Scheduled consequences		
4.4 Behavioral experiments	☐		14.1 Behavior cost	☐	
5. Natural consequences			14.2 Punishment	☐	
5.1 Information about health consequences	☐		14.3 Remove reward	☐	
5.2 Salience of consequences	☐		14.4 Reward approximation	☐	
5.3 Information about social and environmental consequences	☐		14.5 Rewarding completion	☑	
5.4 Monitoring of emotional consequences	☐		14.6 Situation-specific reward	☐	
5.5 Anticipated regret	☐		14.7 Reward incompatible behavior	☐	
5.6 Information about emotional consequences	☐		14.8 Reward alternative behavior	☐	
6. Comparison of behavior			14.9 Reduce reward frequency	☐	
6.1 Demonstration of the behavior	☐		14.10 Remove punishment	☐	
6.2 Social comparison	☑		15. Self-belief		
6.3 Information about others' approval	☐		15.1 Verbal persuasion about capability	☐	
7. Associations			15.2 Mental rehearsal of successful performance	☐	
7.1 Prompts/cues	☑	Personally set health reminders	15.3 Focus on past success	☐	
7.2 Cue signaling reward	☐		15.4 Self-talk	☐	
7.3 Reduce prompts/cues	☐		16. Covert learning	☐	
7.4 Remove access to the reward	☐		16.1 Imaginary punishment	☐	

WEARABLE DEVICE: Withings Steel Active

Reviewer: Dr. Koyya, Maddison, Grace

7.5 Remove aversive stimulus	☐		16.2 Imaginary reward	☐	
7.6 Satiation	☐		16.3 Vicarious consequences	☐	
7.7 Exposure	☐		**Functionality**		
7.8 Associative learning	☐		Battery lasts <1 day	☐	
**8 Repetition and substitution**			Battery lasts 1-2 days	☐	
8.1 Behavioral practice/rehearsal	☐		Battery lasts 3-4 days	☐	
8.2 Behavior substitution	☐		Battery lasts 5-6 days	☐	
8.3 Habit formation	✓		Battery last ≥7 days	✓	After 2 weeks, the battery was at 95%
8.4 Habit reversal	☐		Device pairs with a phone/tablet	✓	
8.5 Overcorrection	☐		Device pairs with a computer	☐	
8.6 Generalisation of target behavior	☐		Device syncs with phone/table notifications	✓	
8.7 Graded tasks	☐		Device face activity display	✓	
**9. Comparison of outcomes**			Mobile app activity display	✓	
9.1 Credible source	☐		Computer activity display	☐	
9.2 Pros and cons	☐		Wrist worn	✓	
9.3 Comparative imagining of future outcomes	☐		Other wear options	☐	
**10. Reward and threat**			**Behavior monitoring**		
10.1 Material incentive (behavior)	☐		Sleep	✓	
10.2 Material reward (behavior)	☐		Nutrition	✓	
10.3 Non-specific reward	✓	Virtual badges	Sedentary	✓	
10.4 Social reward	☐		Exercise (workout tracking)	✓	

Physical activity behaviors tracked:

Steps per Day	✓	Minutes per Day (total)	✓	Exercise minutes per Day	✓	Energy expenditure (total)	✓	Exercise energy expenditure	✓
Sitting (idle) time	☐	Heart rate	✓	Other: Pregnancy tracking	✓	Other: Blood pressure	✓	Other: distance	✓
