# Supplementary material for: Analysis of the Behavioral Change and Utility Features of Electronic Activity Monitors
Source: Technologies (Basel). Author manuscript; Available in PMC 2025 Jan 28. (PMC11774501; doi:10.3390/technologies8040075)
Supplement: Additional File 3 [file NIHMS2016016-supplement-Additional_File_3.pdf]

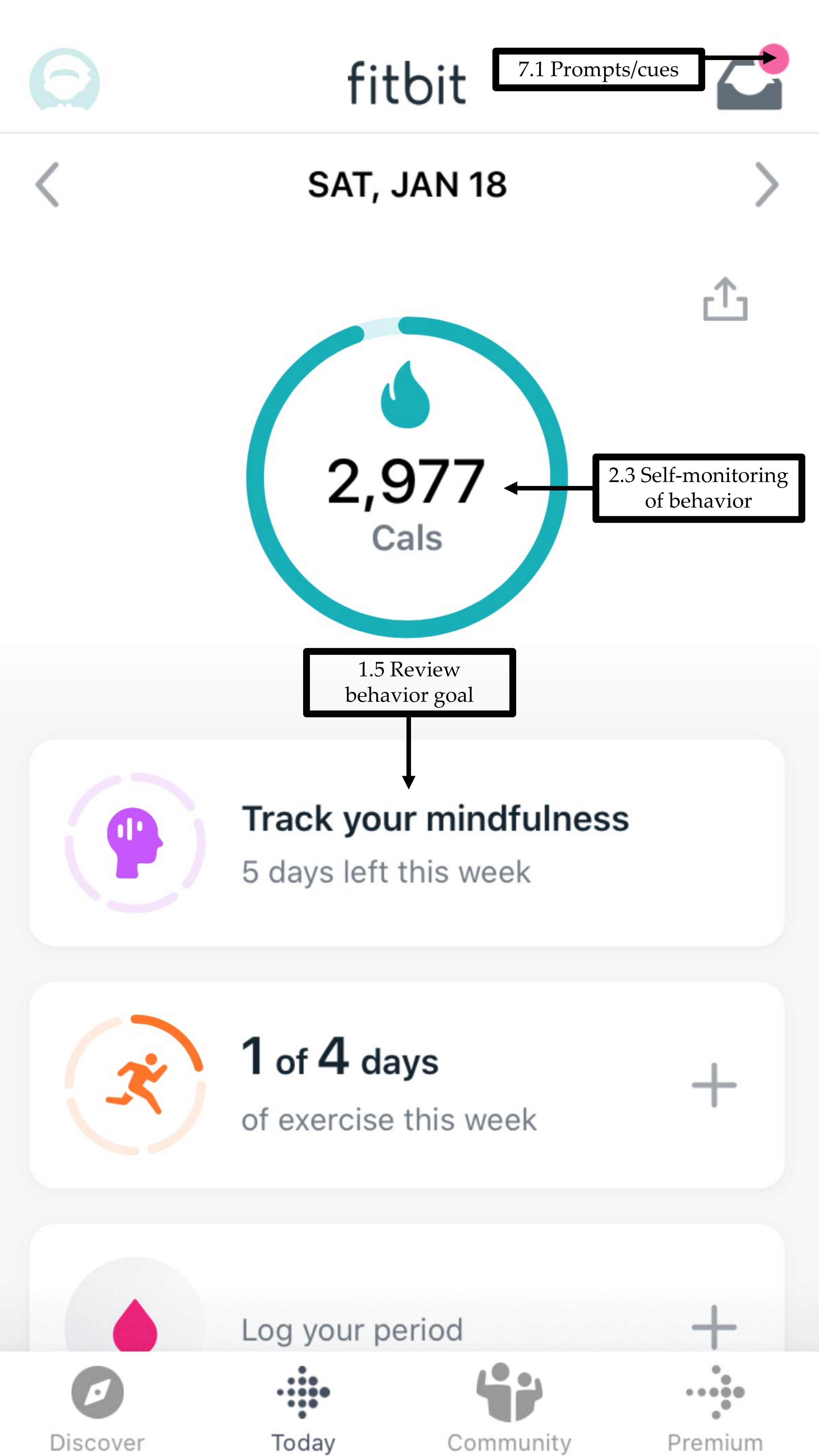
fitbit
7.1 Prompts/cues
SAT, JAN 18
2,977
Cals
2.3 Self-monitoring of behavior
1.5 Review behavior goal
Track your mindfulness
5 days left this week
1 of 4 days
of exercise this week
Log your period
Discover
Today
Community
Premium

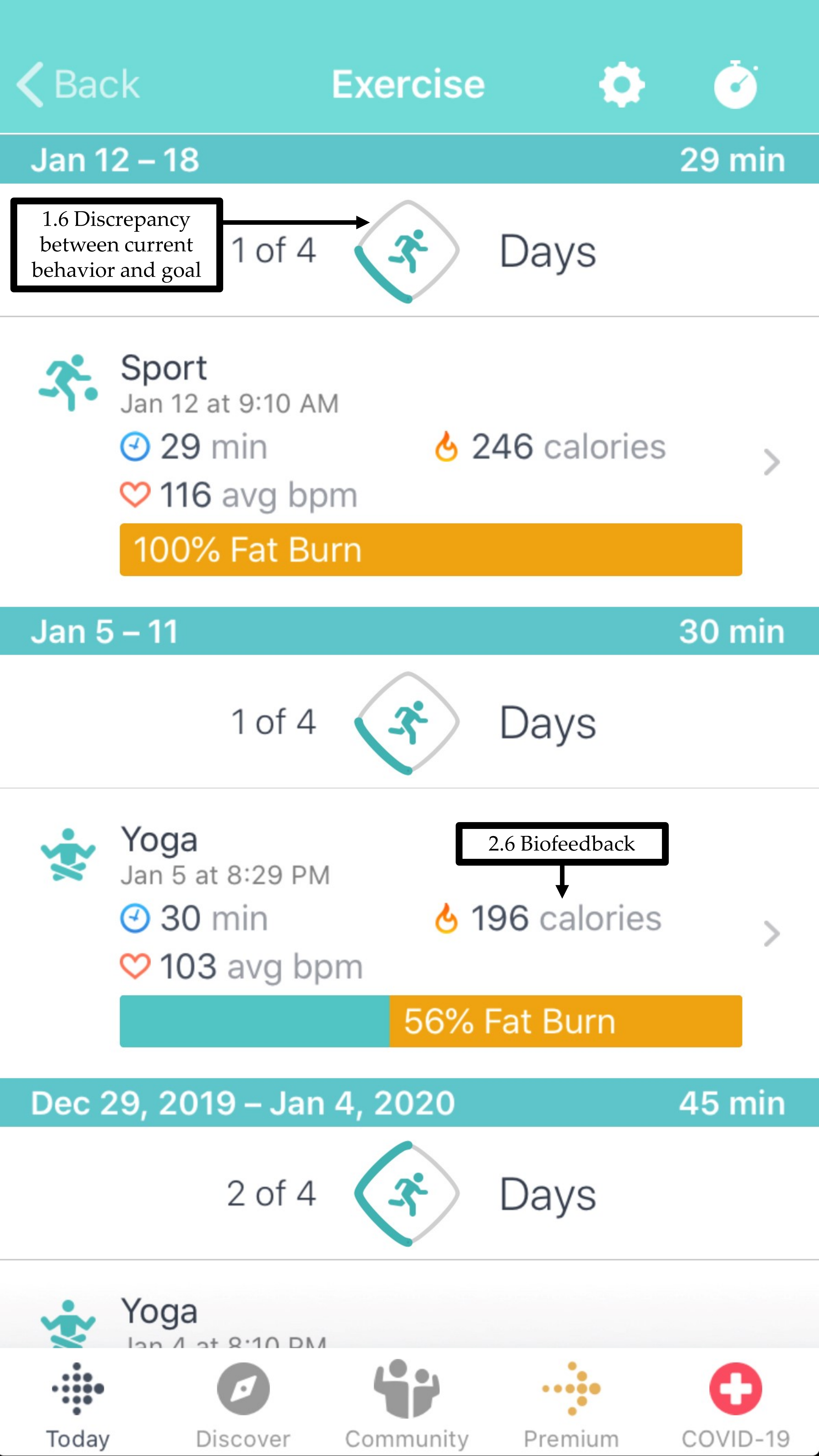
Back
Exercise
Jan 12 – 18
29 min
1.6 Discrepancy between current behavior and goal
1 of 4
Days
Sport
Jan 12 at 9:10 AM
29 min
246 calories
116 avg bpm
100% Fat Burn
Jan 5 – 11
30 min
1 of 4
Days
Yoga
2.6 Biofeedback
Jan 5 at 8:29 PM
30 min
196 calories
103 avg bpm
56% Fat Burn
Dec 29, 2019 – Jan 4, 2020
45 min
2 of 4
Days
Yoga
Today
Discover
Community
Premium
COVID-19

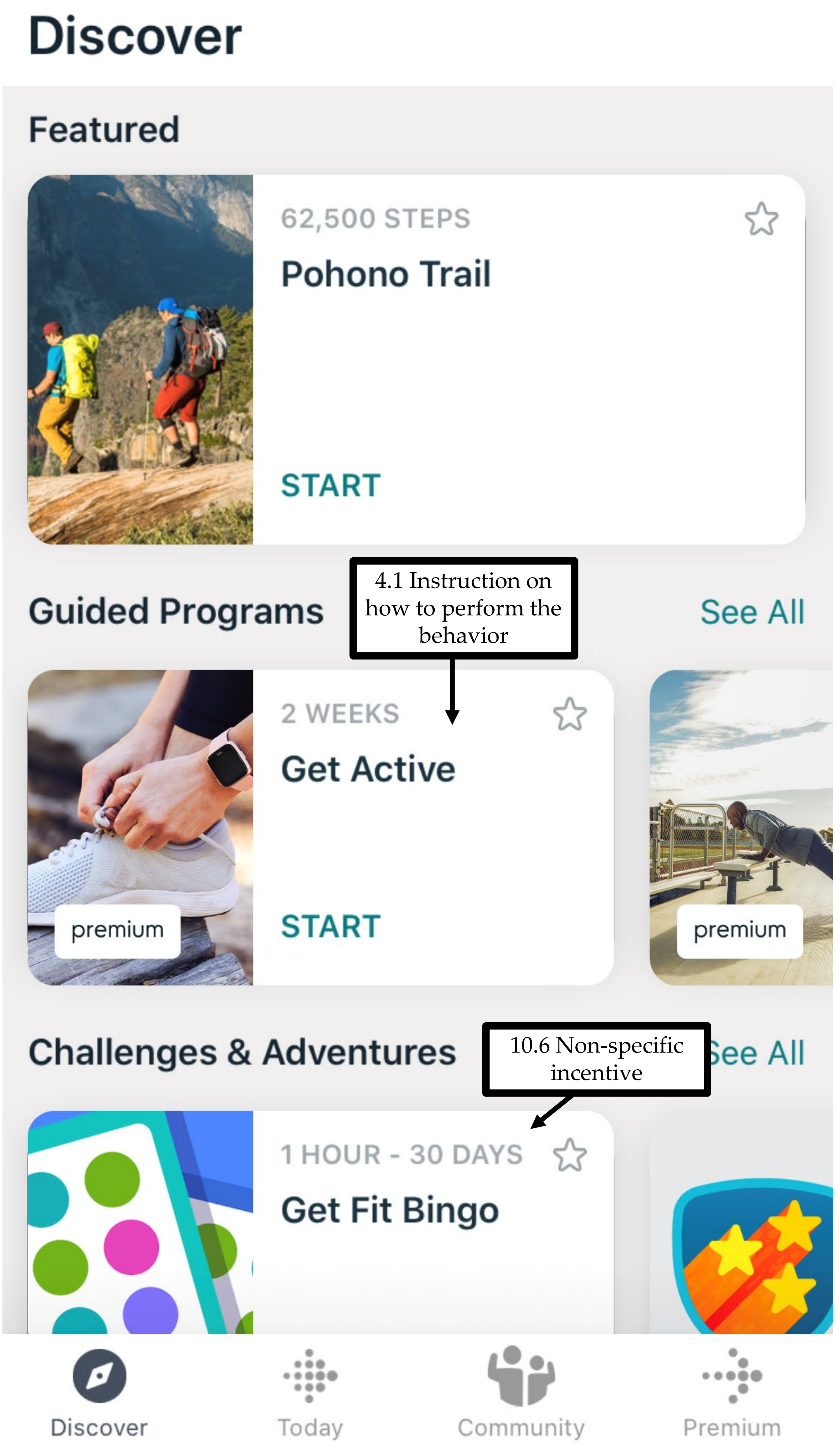
Discover
Featured
62,500 STEPS
Pohono Trail
START
Guided Programs
4.1 Instruction on how to perform the behavior
See All
2 WEEKS
Get Active
START
premium
premium
Challenges & Adventures
10.6 Non-specific incentive
See All
1 HOUR - 30 DAYS
Get Fit Bingo
Discover
Today
Community
Premium

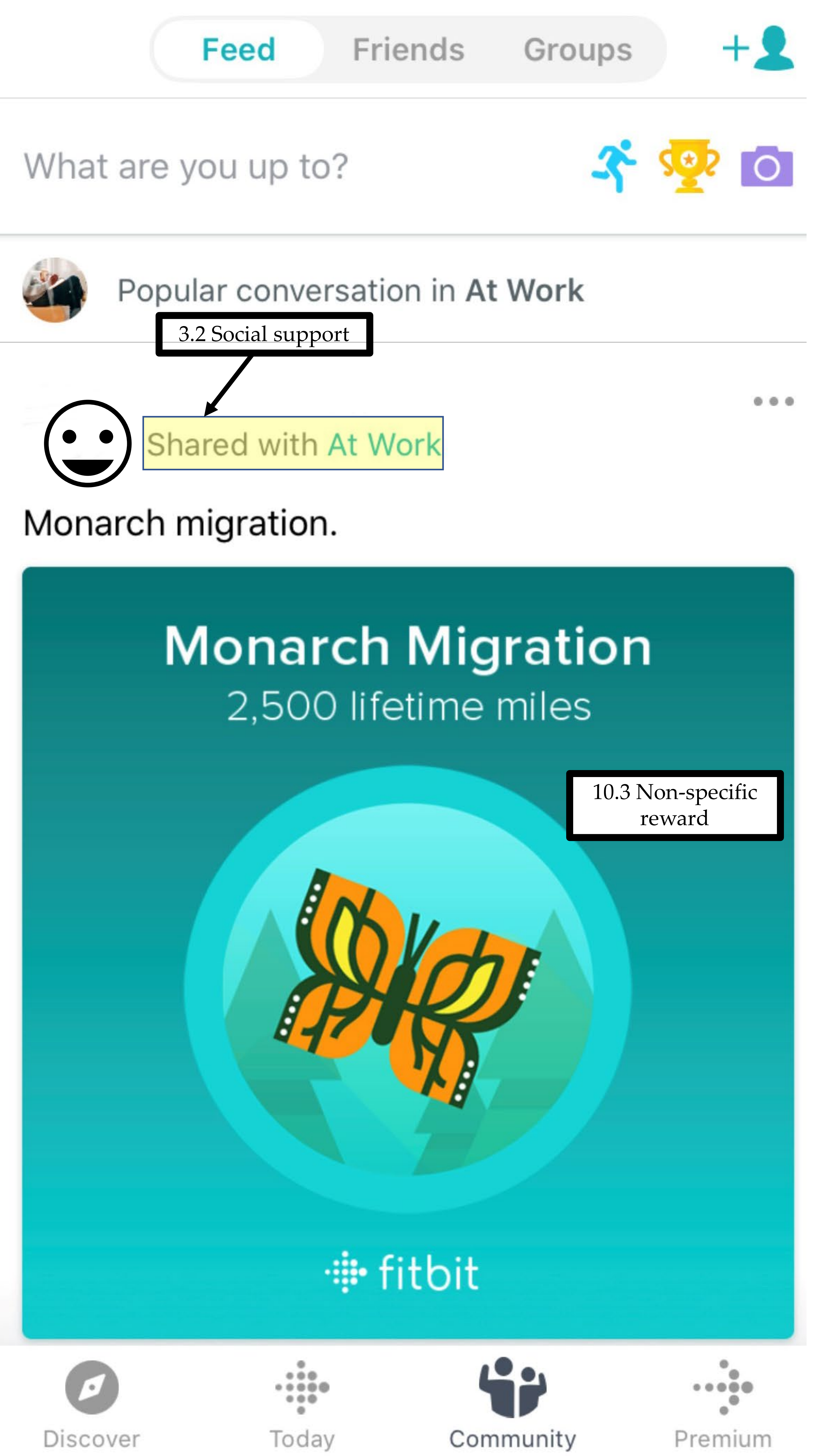
Feed
Friends
Groups
What are you up to?
Popular conversation in At Work
3.2 Social support
Shared with At Work
Monarch migration.
Monarch Migration
2,500 lifetime miles
10.3 Non-specific reward
fitbit
Discover
Today
Community
Premium
